# Latrophilin-2 is a novel receptor of LRG1 that rescues vascular and neurological abnormalities and restores diabetic erectile function

**DOI:** 10.1038/s12276-022-00773-5

**Published:** 2022-05-13

**Authors:** Guo Nan Yin, Do-Kyun Kim, Ji In Kang, Yebin Im, Dong Sun Lee, Ah-reum Han, Jiyeon Ock, Min-Ji Choi, Mi-Hye Kwon, Anita Limanjaya, Saet-Byel Jung, Jimin Yang, Kwang Wook Min, Jeongwon Yun, Yongjun Koh, Jong-Eun Park, Daehee Hwang, Jun-Kyu Suh, Ji-Kan Ryu, Ho Min Kim

**Affiliations:** 1 National Research Center for Sexual Medicine and Department of Urology, Inha University School of Medicine, Incheon, 22332 Republic of Korea; 2 Center for Biomolecular and Cellular Structure, Institute for Basic Science (IBS), Daejeon, 34126 Republic of Korea; 3 Korea Zoonosis Research Institute, Jeonbuk National University, Iksan, 54596 Republic of Korea; 4 Graduate School of Medical Science & Engineering, Korea Advanced Institute of Science and Technology (KAIST), Daejeon, 34141 Republic of Korea; 5 School of Biological Sciences, Seoul National University, Seoul, 08826 Republic of Korea

**Keywords:** Biological therapy, Cytokines, Recombinant protein therapy, Extracellular signalling molecules

## Abstract

Diabetes mellitus (DM) is a chronic metabolic disorder characterized by inappropriate hyperglycemia, which causes endothelial dysfunction and peripheral neuropathy, ultimately leading to multiple complications. One prevalent complication is diabetic erectile dysfunction (ED), which is more severe and more resistant to treatment than nondiabetic ED. The serum glycoprotein leucine-rich ɑ-2-glycoprotein 1 (LRG1) is a modulator of TGF-β-mediated angiogenesis and has been proposed as a biomarker for a variety of diseases, including DM. Here, we found that the adhesion GPCR latrophilin-2 (LPHN2) is a TGF-β-independent receptor of LRG1. By interacting with LPHN2, LRG1 promotes both angiogenic and neurotrophic processes in mouse tissue explants under hyperglycemic conditions. Preclinical studies in a diabetic ED mouse model showed that LRG1 administration into the penile tissue, which exhibits significantly increased LPHN2 expression, fully restores erectile function by rescuing vascular and neurological abnormalities. Further investigations revealed that PI3K, AKT, and NF-κB p65 constitute the key intracellular signaling pathway of the LRG1/LPHN2 axis, providing important mechanistic insights into LRG1-mediated angiogenesis and nerve regeneration in DM. Our findings suggest that LRG1 can be a potential new therapeutic option for treating aberrant peripheral blood vessels and neuropathy associated with diabetic complications, such as diabetic ED.

## Diabetes: Restoring erectile function lost to diabetes

A protein that initiates multiple tissue repair processes could offer effective treatment for erectile dysfunction arising from diabetes mellitus. High blood sugar levels can lead to serious damage to nerves and blood vessels, with impaired male sexual function one potential consequence. Guo Nan Yinof the Inha University School of Medicine, Incheon, South Korea, and colleagues have identified a molecular pathway that can be targeted to reverse such damage. They determined that a protein called LRG1 acts on a receptor called LPHN2 to stimulate blood vessel and nerve growth. LPHN2 is highly expressed in the penile tissue of diabetic humans and rodents, and direct injection of LRG1 effectively restored erectile function in mice. The authors hypothesize that this same pathway could also be used for treatments for other diabetic complications, such as chronic wounds.

## Introduction

Diabetes mellitus (DM) is a chronic metabolic disorder that involves endothelial dysfunction and neuropathy and can lead to multiple complications, including cardiovascular disease, stroke, chronic kidney disease, foot ulcers, and retinopathy^1^. DM is also a major cause of erectile dysfunction (ED), which is a manifestation of microangiopathy and neuropathy^1^. Approximately 50–75% of male diabetic patients have ED^2^. Phosphodiesterase type 5 (PDE5) inhibitors are the most commonly used first-line treatment options for ED; however, these agents are ineffective in ~30% of patients and ultimately cannot rescue angiopathy and neuropathy in diabetic ED patients^3^. Tested alternative therapeutic options for ED include various angiogenic or neurotrophic factors, such as COMP-Ang1, vascular endothelial growth factor (VEGF), dickkopf2, neurotrophin-3 (NT3), and brain-derived neurotrophic factor (BDNF)^4–8^; however, these treatments have shown limited success in clinical trials.

Leucine-rich ɑ-2-glycoprotein 1 (LRG1) is a secreted glycoprotein that is abundant in serum (10–50 μg/mL)^9^. LRG1 has received attention as a prognostic/diagnostic biomarker for many diseases, including inflammatory disease^10,11^, heart failure^12^, neurodegenerative disease^13,14^, and several types of cancer^15–18^. Moreover, it has also been reported that LRG1 is associated with several types of diabetic complications^19–21^. However, little is known about the relationship between LRG1 and diabetic ED, and the molecular mechanisms for the multifunctional role of LRG1 in patients with DM complications remain to be established. Conversely, local administration of recombinant LRG1 was reported to promote corneal re-epithelialization and nerve regeneration in a diabetic mouse model^22^ and to accelerate keratinocyte migration and skin wound repair in normal mice^23^. Moreover, LRG1 overexpression reportedly improves glucose homeostasis and delays the diabetic phenotype in obese mice^24^.

LRG1 promotes angiogenesis by modulating endothelial transforming growth factor (TGF)-β signaling^25^. In the presence of TGF-β, LRG1 directly binds to the TGF-β accessory receptor endoglin (ENG), thereby switching the angiostatic TGF-β signaling (ALK5-Smad2/3 pathway) toward pro-angiogenic TGF-β signaling (ALK1-Smad 1/5/8 pathway). However, compared with typical receptor-ligand interactions, LRG1 shows relatively weak binding affinity for ENG (~2.9 μM), raising questions of whether an additional unknown component is required for TGF-β receptor activation or if LRG1 signals through another mechanism.

Here, we used ligand-based receptor capture technology on living cells and discovered that the adhesion G-protein-coupled receptor latrophilin-2 (LPHN2) is a TGF-β-independent receptor of LRG1. We found that LRG1 acts through LPHN2 to induce both angiogenic and neurotrophic processes in hyperglycemia. Moreover, LPHN2 expression is significantly increased in the penile tissues of a streptozotocin (STZ)-induced diabetic model mice, as well as diabetic ED patients. Accordingly, intra-cavernous administration of recombinant LRG1 ameliorates vascular and neurological abnormalities, thus effectively restoring erectile function. These findings provide insight into the TGF-β-independent mechanism of LRG1 as an angioneurin in hyperglycemia and its potential therapeutic application against various diabetic complications associated with endothelial dysfunction and neuropathy.

## Materials and Methods

### Cell culture

Human umbilical vein endothelial cells (HUVECs; Cat# CC-2519, Lonza) and human embryonic kidney 293 T cells (HEK293T; Cat# CRL-3216, ATCC) were authenticated according to ATCC guidelines. Cells were maintained at 37 °C under humidified 5% CO_2_ conditions. Cells between passages 2 and 7 were used for all experiments. Primary mouse cavernous endothelial cells (MCECs) were prepared and maintained in complete M199 as described previously^26–28^. Diabetes-induced angiopathy was mimicked by serum-starving the cells overnight and exposing them to high-glucose (30 mM glucose) conditions for 72 h at 37 °C in a humidified 5% CO_2_ atmosphere^29^. Normal glucose (5 mM glucose, Cat# G7021, Sigma–Aldrich) conditions were used as a control.

### Animals

Eight-week-old male C57BL/6 mice (Orient Bio, Korea) and LRG1-Tg male mice were used in this study. Transgenic mice with whole-body overexpression of LRG1 were generated by Macrogen (Seoul, Korea) using a construct in which the full LRG1 ORF was subcloned between the RsrII and Notl restriction sites of the pCMV6-Kan/Neo vector (#PCMV6KN, OriGene Technologies Inc.). All manipulations were conducted with the approval of Macrogen’s Institutional Animal Care and Use Committee. The incorporation of the gene in transgenic offspring was screened by PCR analysis of genomic DNA using genotyping primers (CMV_LRG1_F: 5’– ACCATGGTGATGCGGTTTTG-3’; CMV_LRG1_R: 5’–CACCGACAGCTGGACAGTGT-3’). In all experiments, mice were age-matched, and wild-type littermates were used as controls. Experiments were conducted with the approval of the Inha University Animal Care and Use Committee (Assurance Number: INHA 180523-570). Diabetes was induced by intraperitoneal injection of streptozotocin (STZ; 50 mg/kg body weight) for five consecutive days as described previously^30^. Eight weeks after diabetes induction, the mice were anesthetized with intramuscular injections of ketamine (100 mg/kg) and xylazine (5 mg/kg).

### Recombinant protein expression and purification

For recombinant LRG1 protein expression, human LRG1 (residues V36-Q347) was cloned into a modified pcDNA3.1 vector encoding a thrombin recognition sequence followed by the Fc domain of human IgG. Recombinant LRG1 protein was transiently expressed in Freestyle 293-F cells (Invitrogen). After 4 days of culture, Fc-fused LRG1 protein was purified with protein A resin (Cat# 1010025, Amicogen). Tag-free human LRG1 was eluted after on-resin thrombin digestion (0.5% [v/v] with 20 mM Tris-HCl pH 8.0, 200 mM NaCl) at 4 °C overnight. To express recombinant Fc-tagged LPHN2 ectodomain variants, the residues F26-Q95 (Lec domain), V135-P394 (Olf domain), F26-P394 (Lec-Olf domains) or F26-R796 (Lec-Olf-GAIN/GPS domains) were respectively cloned into a modified pcDNA3.1 vector, which encodes a thrombin recognition sequence followed by the Fc domain of human IgG. YFP-His-tagged human LRG1 (LRG1-YFP) and Fc-tagged LPHN2 ectodomain variants (Lec, OlF, Lec-Olf, or Lec-Olf-GAIN/GPS) were produced using an Expi293F expression system (Thermo Fisher) according to the manufacturer’s instructions. After 2 days of culture, LRG1-YFP and Fc-tagged LPHN2 ectodomain variants were purified with Ni-NTA resin (Cat# 30230, Qiagen) and protein A resin (Cat# 1010025, Amicogen), respectively. Tag-free LPHN2 ectodomain variants were eluted after on-resin thrombin digestion (0.5% [v/v] in 20 mM Tris-HCl pH 8.0, 200 mM NaCl) at 4 °C overnight. The eluted LRG1, LRG1-YFP and LPHN2 ectodomain proteins were further purified by a Superdex 200 Increase 10/300 GL column (GE Healthcare Life Sciences).

### Tube formation assay and cell migration assay

Tube formation assays were performed as described previously^28^. Tube formation was monitored for 12–16 h under a phase-contrast microscope and quantified by counting the number of master junctions in four separate experiments in a blinded manner using ImageJ software. For Transwell migration assays, 1 × 10^5^ HUVECs in 200 μl of serum-free M199 were seeded into the upper inserts (8-μm pores) of 12-well plates (Cat# 353182, Corning), and 600 μl of M199 supplemented with 20% FBS was added to the lower chamber. HUVECs or MCECs were then treated with 1 μg/ml PBS (control), 1 μg/ml LRG1, 5 ng/ml TGF-β1 (Cat# 240-B, R&D Systems), 10 μg/ml anti-TGF-β1 antibody (Cat# MAB1835, R&D Systems), and 5 ng/ml TGF-β1 + 10 μg/ml anti-TGF-β1 antibody (premixed overnight for neutralization). After 18 h of culture at 37 °C, non-migrated cells on the upper surface of the insert were removed with a cotton swab. Migrated cells were fixed and stained with crystal violet (Cat# V5265, Sigma) and then eluted with 10% acetic acid. The number of migrated or invaded cells was counted in four random fields.

### TriCEPS-based ligand–receptor capture (LRC-TriCEPS)

To identify the putative receptor of LRG1, TriCEPS-based ligand–receptor capture (LRC-TriCEPS; cat# P05203, Dualsystems Biotech) was used according to the manufacturer’s directions. Briefly, 300 μg of recombinant LRG1 protein or transferrin (control ligand) was dissolved in 150 μl of 25 mM HEPES buffer (pH 8.2), after which 1.5 μl of the TriCEPS reagent was added to each sample and incubated for 90 min at 22 °C with constant agitation. HEK293T cells (1.2 × 10^8^) in PBS (pH 6.5) were mildly oxidized by incubation with 1.5 mM NaIO_4_ at 4 °C in the dark for 15 min with gentle rotation (Supplementary Fig. 1a). Cells were then washed twice at 300 × g for 5 min and resuspended in two separate tubes containing 18 ml of PBS with 1% FBS (pH 6.5), after which 150 μl of TriCEPS-coupled transferrin or TriCEPS-coupled LRG1 was added to each tube and incubated for 90 min at 4 °C with constant, gentle agitation. After the coupling reaction was completed, the cells were collected, and the cell pellets were sent to dual systems for LC–MS/MS analysis.

### Lentiviral shRNA delivery

After the animals were anesthetized with intramuscular injections of ketamine (100 mg/kg) and xylazine (5 mg/kg), the scrambled control shRNA (shCon) lentivirus particles (Cat# SC-108080, Santa Cruz) or SMART vector mouse lentivirus containing shRNA targeting LPHN2 (shLPHN2; Cat# V3SM7603-234963095, Dharmacon) were administered by intramuscular injection at a dose of 1 × 10^5^ transforming units (TU) per mouse. For in vitro (HUVECs, HEK293T cells and MCECs) and ex vivo (MPGs and DEGs) cell culture experiments, shCon or shLPHN2 lentivirus particles were added to the culture medium at a concentration of 5 × 10^4^ TU/ml. The shRNA sequence targeting murine LPHN2 was ACGGCTTATGGGTCATTTA, and the shRNA sequence targeting human LPHN2 was TCAACTGCTAGGTCGATAT. Experiments were performed 3 days after lentiviral infection.

### Cell surface binding assay

HEK293T cells and HUVECs were plated on 8-well Lab-Tek chamber slides in standard growth medium. The cells were incubated with LRG1-YFP or YFP for 1 h at 37 °C in a humidified 5% CO_2_ environment and then washed twice with PBS and fixed with 4% formaldehyde in PBS for 15 min at room temperature. The wells were washed twice with PBS, and then the slides were mounted with ProLonged Antifade mounting solution (Cat# P10144, Molecular Probes). Fluorescently labeled LRG1 was observed under a confocal laser-scanning microscope (Zeiss LSM 780).

### LC–MS/MS analysis of immunoprecipitants

HUVECs were serum-starved for 6 h and then treated with LRG1 (1 µg/ml) for 0, 10, 30, and 60 min. After being lysed with RIPA buffer (Cat# 89900, Sigma), total cell lysates were immunoprecipitated with LPHN2 antibody (Cat# sc-514197, Santa Cruz; 1:50) and analyzed by SDS–PAGE and Coomassie Blue staining. The indicated bands (Supplementary Fig. 1b, framed in red) were excised from the SDS–PAGE gels, and Nano LC–MS/MS analyses were performed using an Easy n-LC (Thermo Fisher, San Jose, CA, USA) and an LTQ Orbitrap XL mass spectrometer (Thermo Fisher) equipped with a nanoelectrospray source^31^ (Yonsei Proteome Research Center, Seoul, Republic of Korea). The Mascot algorithm (Matrix Science, USA) was used to identify peptide sequences present in a protein sequence database. The peptides were filtered with a significance threshold of *P* < 0.05.

### Aortic ring assay

Aortas were harvested from 8-week-old C57BL/6 mice, positioned in the 8-well Nunc Lab-Tek Chamber Slide System (Sigma–Aldrich) and kept in place with an overlay of 50 µl of Matrigel. Aortic rings were cultured in complete M199 for 5 days in normal glucose (5 mM) or high-glucose (30 mM) medium, with or without LRG1 (1 µg/ml), shCon or shLPHN2 (5 × 10^4^ TU/ml culture medium). Aortic segments and sprouting cells were fixed in 4% paraformaldehyde for at least 30 min and used for immunofluorescence analysis of PECAM-1 (Cat# MAB1398Z, Millipore; 1:50)^32^.

### Ex vivo endothelial cell sprouting assay

Mouse corpus cavernosum tissue was cut into two or three pieces and then plated on Matrigel stock solution-coated 6-well cell culture dishes. The Matrigel was polymerized by incubating for 15 min at 37 °C, and 3 ml of normal-glucose (5 mM) or high-glucose (30 mM) conditioned complete M199, with or without 1 µg/ml LRG1 and shRNA (shCon or shLPHN2; 5 × 10^4^ TU/ml culture medium), was added to the cell culture dish. The cells were then incubated at 37 °C in a humidified 5% CO_2_ environment, and the conditioned complete M199 was changed every 2 days. Images were obtained with a phase-contrast microscope after 7 days, and the sprouting cell density was analyzed using ImageJ 1.34.

### MPG and DRG explant culture and sprouting assay

Mouse major pelvic ganglion (MPG) and dorsal root ganglion (DRG) tissues were dissected and maintained as described previously^33^. After culture for 7 days under normal-glucose (5 mM) or high-glucose (30 mM) conditions, with or without LRG1 (1 µg/ml) and shRNA (shCon or shLPHN2; 5 × 10^4^ TU/ml culture medium) at 37 °C in a humidified 5% CO_2_ environment, neurite outgrowth segments were fixed in 4% paraformaldehyde for at least 30 min and immunostained with an anti-βIII tubulin antibody (Cat# ab107216, Abcam; 1:100).

### Measurement of erectile function

Erectile function was measured as described previously^30^. The fasting and postprandial blood glucose concentrations in streptozotocin (STZ)-treated WT or LRG1-Tg diabetic mice were significantly higher than those in control or LRG1-Tg mice. In addition, body weight and serum insulin levels were significantly lower in STZ-induced diabetic mice than in controls or LRG1-Tg mice. However, the body weight and blood glucose levels of diabetic mice did not differ significantly regardless of treatment (Supplementary Tables 1–4).

### BrdU labeling

Mice from each group were intraperitoneally injected with BrdU (50 mg/kg body weight; Cat# 19–160, Sigma–Aldrich) once daily for three consecutive days and sacrificed 1 day after the final BrdU injection. An additional antigen retrieval step was performed with 5’-bromo-2’-deoxyuridine antibody (BrdU; Cat. MCA2060, AbD Serotec; 1:50). The number of BrdU-positive endothelial cells was counted in four different regions. The values are expressed per high-power field.

### TUNEL assay

TUNEL (terminal deoxynucleotidyl transferase-mediated deoxyuridine triphosphate nick-end labeling) assays were used to evaluate cell death in cavernous tissue from control and STZ-induced diabetic mice. The ApopTag Fluorescein In Situ Apoptosis Detection Kit (Cat# S7160, Chemicon) was used according to the manufacturer’s instructions. Digital images and the numbers of apoptotic cells were determined using a confocal fluorescence microscope. For in vitro studies, the number and percentage of TUNEL-positive cells were evaluated.

### Histological examination of immunofluorescence-stained tissue

Penile tissue was fixed in 4% paraformaldehyde for 24 h at 4 °C, then frozen and cut into sections (12 μm) for the indicated immunofluorescence experiments. Cultured MPG tissues, DRG tissues, and aortic rings were fixed in 4% paraformaldehyde for 10 min at room temperature and then incubated with blocking solution for 2 h at room temperature. Thereafter, the samples were incubated overnight at 4 °C with the following primary antibodies: anti-LPHN2 (Cat# ab138498, Abcam; 1:100), anti-PECAM-1 (Cat# MAB1398Z, Millipore; 1:50), anti-NG2 (Cat# AB5320, Millipore; 1:50), anti-βIII tubulin (Cat# ab107216, Abcam; 1:100), anti-LRG1 (Cat# HPA001888, Sigma; 1:100), anti-TGF-β1 (Cat# sc-146, Santa Cruz Biotechnology; 1:100), anti-phospho-eNOS (Cat# 9571, Cell Signaling; 1:100), anti-claudin-5 (Cat# 352500, Thermo Fisher Scientific; 1:100), anti-oxidized LDL (Cat# ab14519, Abcam; 1:100), and anti-nNOS (Cat# sc-5302, Santa Cruz Biotechnology; 1:100). After several washes with PBS, the samples were incubated with tetramethylrhodamine (TRITC)-conjugated donkey anti-chicken secondary antibodies (Cat# 703-025-155, Jackson ImmunoResearch Laboratories; 1:200), fluorescein isothiocyanate (FITC)-conjugated goat anti-Armenian hamster secondary antibodies (Cat# 127-095-160, Jackson ImmunoResearch Laboratories; 1:200), or DyLight 550-conjugated donkey anti-rabbit secondary antibodies (Cat# ab98489, Abcam; 1:200) for 2 h at room temperature. Signals were visualized, and digital images were obtained using a confocal fluorescence microscope (K1-Fluo; Nanoscope Systems, Inc.). Immunofluorescence staining intensity was quantified using ImageJ 1.34.

### Immunoblots and immunoprecipitation

Cells and tissues were lysed in RIPA buffer (Sigma–Aldrich) supplemented with protease inhibitors (Cat# P3100-001, GenDEPOT) and phosphatase inhibitors (Cat# P3200-001, GenDEPOT). Equal amounts of protein (30 µg per lane) from each whole-cell or tissue lysate were resolved by SDS–PAGE on 8 to 12% gels and transferred to polyvinylidene fluoride (PVDF) membranes. After being blocked with 5% non-fat dried milk for 1 h at room temperature, the membranes were incubated at 4 °C overnight with the following primary antibodies: anti-LPHN2 (Cat# ab209548, Abcam; 1:500), anti-LRG1 (Cat# HPA001888, Sigma; 1:500), anti-TGF-β1 (Cat# sc-146, Santa Cruz Biotechnology; 1:500), anti-phospho-eNOS (Cat# 9571, Cell Signaling; 1:500), anti-eNOS (Cat# 610297, Becton Dickinson; 1:1000), and β-actin (Cat# ab8226, Abcam; 1:6000). For immunoprecipitation, 1,000 μg of lysate was incubated with the indicated antibody (1–2 μg) for 3–4 h at 4 °C followed by overnight incubation with Protein A/G PLUS-Agarose (Cat# SC-2003, Santa Cruz Biotechnology). Immunoprecipitates were washed five times with RIPA buffer and then resolved by SDS–PAGE and immunoblotted with the indicated antibodies. TGF-β1 levels in conditioned medium (CM) were measured by first harvesting and centrifuging the CM at 1500 rpm for 5 min to remove cell debris and then precipitating CM samples with a trichloroacetic acid (TCA) and acetone mixture (10% TCA and 10 mM dithiothreitol [DTT] in acetone) at −20 °C overnight. The precipitated proteins were washed twice with 20 nM DTT in acetone and resuspended in RIPA buffer (Sigma–Aldrich) supplemented with protease inhibitors (Cat# P3100-001, GenDEPOT) and phosphatase inhibitors (Cat# P3200-001, GenDEPOT). Equal amounts of protein (30 µg per lane) were resolved by SDS–PAGE and immunoblotted with anti-TGF-β1 (Cat# sc-146, Santa Cruz Biotechnology; 1:500). Densitometric analyses of Western blot bands were performed using ImageJ 1.34.

### Cignal Finder GPCR signaling 10-pathway reporter array and phospho array

For Cignal Finder GPCR signaling 10-pathway reporter array analyses, HUVECs (5 × 10^3^ cells/well) diluted in 80 µl of Opti-MEM supplemented with 5% FBS and 0.1 mM nonessential amino acids were transfected by mixing with the preincubation transfection mixture and then incubated for 24 h at 37 °C in a 5% CO_2_ environment. For the pre-incubation transfection mixture, 0.5 µl of Lipofectamine 2000 (Invitrogen) and reporter DNA constructs (Cignal Finder Reporter Array for GPCR signaling 10-Pathway; CCA-109L-2, Qiagen) in 50 μl Opti-MEM were incubated for 30 min at room temperature. After serum starvation for 4 h, reporter DNA-transfected HUVECs were treated with 1 µg/ml LRG1 for 6 h. The 10 signaling pathway reporters in cells were then analyzed by quantifying luminescence signals using the Cignal 10-Pathway Reporter Array Kit according to the manufacturer’s instructions.

For the phospho array, HUVECs were serum-starved for 8 h and then incubated with anti-TGF-β1 antibody for 1 h, after which HUVECs were stimulated with 1 µg/ml LRG1 for 30 min. Whole-cell lysates of HUVECs were prepared, and aliquots of lysates containing 50 µg of protein were used for Phospho Explorer antibody assays using an antibody array assay kit (Cat# KAS02, Fullmoon Biosystems, Sunnyvale, CA, USA) according to the manufacturer’s instructions. The Phospho Explorer antibody array (Full Moon Biosystems, Inc.) consisted of 1318 antibodies, and each antibody was present as two replicates printed on a coated glass microscope slide, as well as multiple positive and negative controls. Phospho Explorer antibody array experiments and analyses were performed as a custom service by E-Biogen (Ebiogen Inc.)

## Results

### The Adhesion GPCR Latrophilin-2 is a Novel TGF-β-independent Receptor of LRG1

**Fig. 1:**
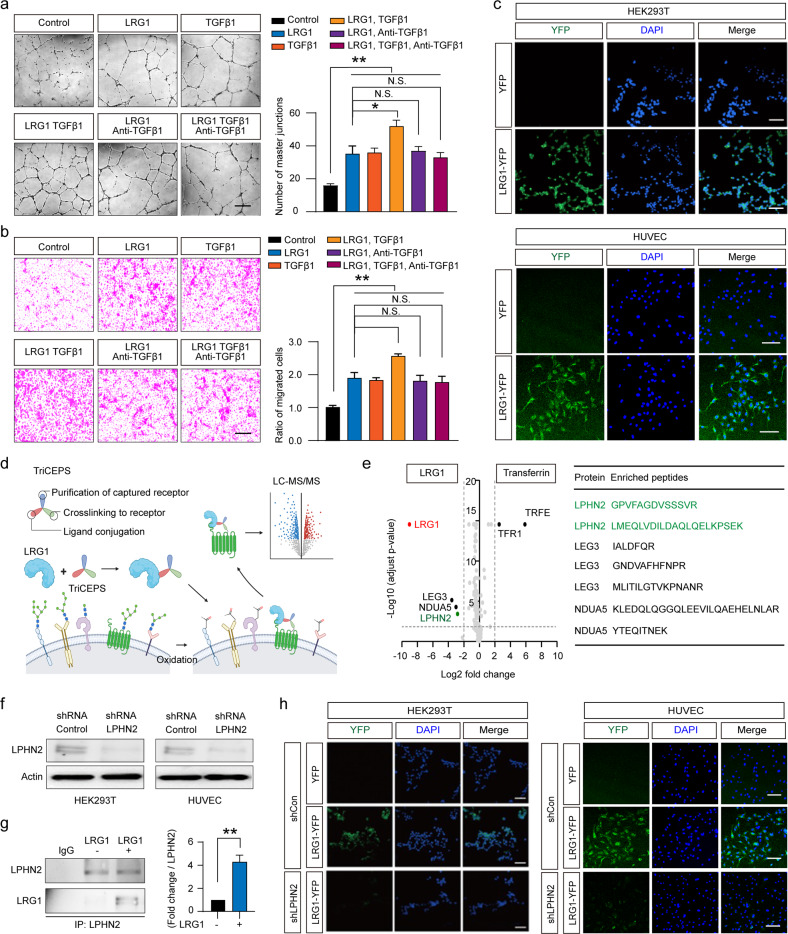
The adhesion GPCR LPHN2 is a TGF-β–independent receptor of LRG1. **a, b** Tube formation assay **(a)** and transwell cell migration assays **(b)** using HUVECs treated with the indicated concentrations of the following proteins: PBS (negative control), LRG1 (1 μg/ml), TGF-β1 (5 ng/ml), LRG1 (1 μg/ml) + TGF-β1 (5 ng/ml), LRG1 (1 μg/ml) + anti-TGF-β1 Ab (10 μg/ml), and LRG1 (1 μg/ml) + TGF-β1 (5 ng/ml) + anti-TGF-β1 Ab (10 μg/ml). Left: Representative images. Scale bars, 200 µm. Right: Master junctions and migrated cells were quantified using ImageJ, and the results are presented as the means ± SEM (*n* = 4). The relative ratio of the control group was defined as 1. **c** Cell surface binding of LRG1-YFP or YFP to HEK293T cells (top) and HUVECs (bottom) was assessed after treatment with LRG1-YFP (10 μg/ml) or YFP (10 μg/ml). Nuclei were labeled with 4,6-diamidino-2-phenylindole (DAPI; blue). Scale bar, 100 µm. **d** Schematic diagram of LRC-TriCEPS technique to identify a TGF-β-independent receptor of LRG1. **e** Volcano plots showing FDR-adjusted *P* values plotted against fold changes between samples comparing TriCEPS-coupled LRG1 or Transferrin with the glycine-quenched TriCEPS reagent control sample. The three proteins LPHN2, LEG3 and NDUA5 represent receptor candidates and were defined as those with an enrichment factor greater than 4 and an FDR-adjusted *P* value < 0.05. **f** LPHN2 expression in HEK293T cells and HUVECs after infection with scramble shRNA control (shCon, 5 × 10^4^ TU/ml) or LPHN2 knockdown shRNA lentivirus (shNPHN2, 5 × 10^4^ TU/ml) was analyzed by Western blotting. **g** Immunoprecipitation (IP) of LPHN2 in whole HUVEC lysates treated with or without LRG1 (1 μg/ml) for 10 min followed by immunoblot analysis to detect LRG1 and LPHN2. Data are the means ± SEM (*n* = 3). **h** Cell surface binding of LRG1-YFP or YFP to control (shCon-expressing) or LPHN2-knockdown (shLPHN2-expressing) HEK293T cells (left) and HUVECs (right) after treatment with LRG1-YFP (10 μg/ml) or YFP (10 μg/ml). Nuclei were stained with DAPI (blue). Scale bar, 100 µm. *P < 0.05; **P < 0.01 (Student’s *t* test). N.S., not significant.

LRG1 has been previously reported to promote angiogenesis in the presence of TGF-β by modulating the endothelial pro-angiogenic Smad1/5/8 signaling pathway^25^. However, we found that LRG1 alone was sufficient to induce tube formation and migration of human umbilical vein endothelial cells (HUVECs), which were cultured under high-glucose conditions to mimic diabetic hyperglycemia (Fig. 1a, b). Furthermore, LRG1-mediated tube formation and migration were unaffected by the presence of TGF-β1-blocking antibody. These findings led us to hypothesize that LRG1 may bind to an unknown receptor and/or activate TGF-β1-independent signaling to induce angiogenesis under hyperglycemic conditions.

We next used a proteomics approach to identify the TGF-β1-independent LRG1 receptor. First, we confirmed that LRG1 bound to the cell surface of the HUVECs and HEK293T cells, indicating that both cell lines expressed an unknown cell surface receptor of LRG1 (Fig. 1c). We then performed ligand-based receptor capture (LRC) in live HEK293T cells, which are amenable to large-scale culture. To this end, we used TriCEPS, a chemoproteomic reagent with three moieties for ligand conjugation, receptor capture, and receptor purification, respectively, which enabled the identification of a glycosylated target receptor^34^. Briefly, HEK293T cells were oxidized with sodium metaperiodate and incubated with TriCEPS-coupled LRG1, and then the captured glycoproteins were affinity purified and identified by liquid chromatography-tandem mass spectrometry (LC–MS/MS) analysis (Fig. 1d, Supplementary Fig. 1a). Based on our selection criteria (enrichment factor > 4 and false discovery rate [FDR]-adjusted *P* value < 0.05), we identified three prospective LRG1 binding partners: galectin-3 (LEG3), NADH dehydrogenase 1 alpha subcomplex subunit 5 (NDUA5), and latrophilin-2 (LPHN2) (Fig. 1e). Of these three proteins, LPHN2 was the only cell membrane protein and was thus the strongest candidate for an LRG1 receptor. Indeed, LPHN2 expression was confirmed in both HUVECs and HEK293T cells (Fig. 1f). LPHN2, also known as calcium-independent alpha-latrotoxin receptor 2 (CIRL2), is an adhesion-type G-protein-coupled receptor (GPCR) that is widely expressed in various tissues; however, its cellular functions are poorly understood^35,36^.

Lentiviral-mediated LPHN2 knockdown in HUVECs and HEK293T cells exhibited significantly decreased cell-surface binding of LRG1-YFP compared to cells treated with scrambled small hairpin RNA (shRNA) control lentivirus (shControl) (Fig. 1f, h). Moreover, LC–MS/MS analysis of HUVECs treated with soluble LRG1 revealed that LPHN2 are co-immunoprecipitated with LRG1, suggesting a specific and direct association of LRG1 with LPHN2 on HUVEC membranes (Fig. 1g, Supplementary Fig. 1b, c). Solid-phase binding assays using LRG1 and the extracellular domains of LPHN2, including lectin (Lec), olfactomedin-like (Olf), and GPCR autoproteolysis-inducing/GPCR proteolysis site (GAIN/GPS) domains, revealed that the Olf domain of LPHN2 was the minimal binding domain for LRG1 (Kd = 920 nM for Olf domain, 890 nM for Lec/Olf domain) (Supplementary Fig. 1d, e). Unlike the Olf domain alone and the Lec/Olf domain, recombinant ecto-full LPHN2 was unable to bind with LRG1. This finding suggested that proper proteolytic processing of the LPHN2 GPS motif at the cellular membrane might also be critical for LRG1 binding and LPHN2-mediated cellular functions^37^. Interestingly, LRG1 could not bind to the Lec/Olf domain of LPHN1 or LPHN3, despite their 87% sequence similarity to the Lec/Olf domain of LPHN2 (Supplementary Fig. 1d, e). These findings demonstrated that LPHN2 is a novel TGF-β-independent receptor of LRG1.

### The LRG1/LPHN2 Axis Induces Microvessel Sprouting and Neurite Outgrowth in a TGF-β-independent Manner Under High-Glucose Conditions

**Fig. 2:**
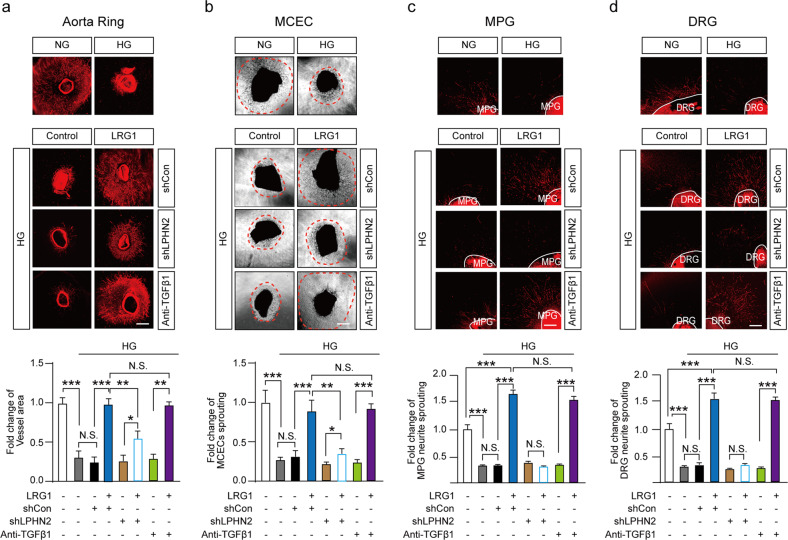
LRG1 and LPHN2 mediate microvessel sprouting and neurite outgrowth in the absence of TGF-β1 under hyperglycemic conditions. **a**, **b** Ex vivo mouse aortic ring assay (**a**) and mouse cavernosum endothelial cell (MCEC) sprouting assay (**b**) under normal-glucose (NG) or high-glucose (HG) conditions with treatment of PBS (negative control) or LRG1 (1 μg/ml) in the presence of lentivirus (shCon or shLPHN2; 5 × 10^4^ TU/ml) or anti-TGF-β1 antibody (10 μg/ml) for 5 days. Sprouted microvessels were immunostained with the endothelial cell marker platelet/endothelial cell adhesion molecule-1 (PECAM-1) (red, **a**). The dotted line indicates the sprouting endothelial cell range (**b**). Scale bars, 100 μm. The intensity of the area of microvessel sprouting from aortic rings (means ± SEM (n = 4) (**a**) and endothelial cell sprouting from cavernous tissue (means ± SEM (*n* = 6) (**b**) were quantified (bottom). **c** and **d** βIII-Tubulin immunostaining in mouse MPG tissue (**c**, top) and DRG tissue (**d**, top) under normal glucose (NG) or high glucose (HG) conditions with treatment of PBS (negative control) or LRG1 (1 μg/ml) in the presence of lentivirus (shCon or shLPHN2; 5 × 10^4^ TU/ml) or anti-TGF-β1 antibody (10 μg/ml) for 1 week. Scale bars, 100 µm. Quantification of βIII-tubulin–immunopositive neurite length in MPG (**c**) and DRG (**d**) tissue and (bottom, means ± SEM (*n* = 4)). *P < 0.05; **P < 0.01; ***P < 0.001 (Student’s *t* test). N.S., not significant. The relative ratio of the NG group was defined as 1.

Endothelial dysfunction in DM manifests as impaired proliferation and migration of endothelial cells (ECs)^1^. As expected, we found that high-glucose conditions impaired in vitro tube formation of mouse cavernous endothelial cells (MCECs) and decreased microvessel sprouting from aortic rings and penile cavernosum tissues (Fig. 2a, b, top; Supplementary Fig. 2a). LRG1 treatment completely rescued all of these high-glucose-induced phenotypes, and lentiviral-mediated LPHN2 knockdown significantly reduced these effects of LRG1 (Fig. 2a, b; Supplementary Fig. 2a, b). Consistent with previous reports^38^, we found slightly increased levels of secreted TGF-β1 in high-glucose culture medium conditioned by MCECs, aortic rings, and penile cavernosum tissues (Supplementary Fig. 2c). However, even in the presence of TGF-β1-blocking antibody, LRG1 effectively promoted micro-vessel sprouting under hyperglycemic conditions (Fig. 2a, b).

Notably, under high-glucose culture conditions, MCECs showed significantly increased LPHN2 expression (Supplementary Fig. 2d), as did major pelvic ganglion (MPG; mixed parasympathetic and sympathetic ganglion that innervates the pelvic organs) and dorsal root ganglion (DRG; a cluster of cell bodies that transmit sensory information to the brain). Additionally, it has been reported that hyperglycemia leads to impairments of Schwann cell proliferation and migration and of axon regeneration from DRG neurons, which are broadly associated with diabetic peripheral neuropathy^39^. Therefore, we next tested whether LRG1 affects neurite outgrowth in MPG and DRG under hyperglycemic conditions. To this end, we exposed MPG and DRG mouse explants to hyperglycemia and analyzed neuronal tissues using βIII-tubulin immunofluorescence staining. In line with a previous report, high-glucose conditions significantly reduced neurite sprouting in both MPG and DRG explants (Fig. 2c, d, top). Importantly, LRG1 treatment stimulated neurite outgrowth from MPG and DRG explants under hyperglycemic conditions, and this effect was abolished by lentiviral-mediated LPHN2 knockdown (Fig. 2c, d). Although high-glucose conditions increased TGF-β1 levels in both MPG and DRG explant cultures, the TGF-β1-blocking antibody did not impact LRG1-mediated neurotrophic effects (Fig. 2c, d, Supplementary Fig. 2c). Taken together, our data showed that TGF-β-independent binding of LRG1 to LPHN2 promotes micro-vessel sprouting and neurite outgrowth under hyperglycemic conditions.

### LRG1 Treatment Restores Erectile Function in Diabetic Mice Through the LPHN2 Receptor

**Fig. 3:**
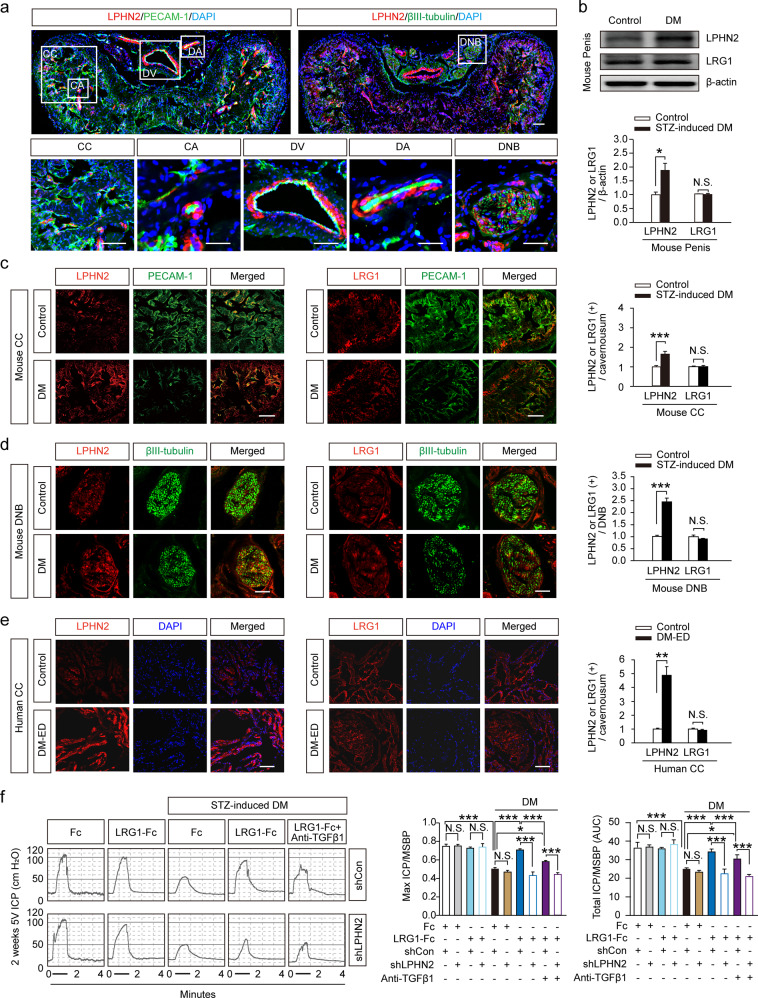
LPHN2 expression is increased in penile tissue of diabetic mouse model, and intracavernous administration of LRG1 ameliorates diabetic erectile dysfunction. **a** Penile tissue from normal mice was cross-sectioned for immunofluorescence staining. Representative LPHN2 (red), PECAM-1 (green, top left) and βIII-Tubulin (green, top right) staining. Nuclei were stained with DAPI (blue). Scale bars, 200 µm. Higher magnification images of the corpus cavernosum (CC; scale bars, 100 µm), cavernous artery (CA; scale bars, 25 µm), dorsal vein (DV; scale bars, 50 µm), dorsal artery (DA; scale bars, 25 µm) and dorsal nerve bundles (DNB; scale bars, 25 µm) (bottom). **b** Representative Western blots showing LPHN2 and LRG1 in mouse cavernous tissue from control and STZ-induced diabetic mice (top). Normalized band intensity values were quantified by ImageJ and are presented as the means ± SEM (*n* = 4). **c** and **d** LPHN2 and LRG1 expression in CC (**c**) and DNB (**d**) tissue from control and STZ-induced diabetic mice (DM). Immunostaining for LPHN2 (red, left), LRG1 (red, middle) with PECAM-1 (**c**, green) or βIII-Tubulin (**d**, green). Scale bars, 100 μm. Immunopositive areas of LPHN2 and LRG1 in mouse CC **(c)** and DNB **(d**) were quantified using ImageJ and are presented as the means ± SEM (*n* = 6, right). **e** LPHN2 and LRG1 expression in human CC tissue from control and diabetic patients with erectile dysfunction (DM-ED). Immunostaining for LPHN2 (left, red) and LRG1 (middle, red). Nuclei were stained with DAPI (blue). Scale bars, 100 μm. Quantification of LPHN2- and LRG1-immunopositive areas in human CC using ImageJ (means ± SEM (*n* = 6, right). **f** Representative intracavernous pressure (ICP) responses in control and STZ-induced diabetic mice at 2 weeks after repeated intracavernous injections of Fc (negative control, 5 µg/20 µl), LRG1-Fc (5 µg/20 µl), or LRG1-Fc (5 µg/20 µl) + anti-TGF-β1 antibodies (10 μg/ml) on Days 0 and 3 with scramble shRNA control (shCon) or LPHN2 knockdown shRNA (shLPHN2) conditions (5 × 10^5^ TU/mouse). The cavernous nerve was stimulated with 5 V. The stimulus interval is indicated by a solid bar. The ratios of mean maximal ICP (**f**, middle) and total ICP (**f**, right) (area under the curve) to mean systolic blood pressure (MSBP) were calculated for each group (*n* = 5). *P < 0.05; **P < 0.01; ***P < 0.001 (Student’s *t* test). N.S., not significant. The relative ratio of the control group was defined as 1.

When DM is not well controlled, hyperglycemia can irreversibly damage the cavernous nerve and blood vessels, leading to ED^1^. However, little is known about the relationship between LRG1 and diabetic ED. Since the interaction of LRG1 with LPHN2 promoted endothelial cell proliferation and neurite outgrowth of MPG and DRG explants under hyperglycemic conditions, we investigated whether LPHN2 is expressed in cavernous tissue. Immunofluorescent staining with anti-LPHN2 antibodies revealed high LPHN2 expression in the corpus cavernosum (CC), dorsal vein (DV), cavernous artery (CA), dorsal artery (DA), and dorsal nerve bundles (DNBs) (Fig. 3a). Interestingly, in the STZ-induced diabetic mouse model (hereafter referred to as diabetic mice), LPHN2 expression was further increased in all penile tissues, particularly in the CC and DNB of penile tissues, while LRG1 levels were not changed (Fig. 3b–d). These findings were consistently observed in cavernosum tissues of human diabetic ED patients (Fig. 3e) compared to tissues from healthy individuals. As an indication of erectile dysfunction, diabetic mice showed profoundly decreased numbers of endothelial cells in cavernosum tissue and neurofilaments in DNBs (Fig. 3c, d).

We previously reported that intra-cavernous delivery of Ninjurin1-neutralizing antibody or Dickkopf2 restored erectile function in diabetic mice through enhanced penile angiogenesis and neural regeneration^6,40^. Therefore, we investigated the therapeutic relevance of the LRG1/LPHN2 axis by evaluating erectile function in diabetic mice after intracavernous injection of LRG1. Upon electrical stimulation of the cavernous nerve, diabetic mice showed significantly decreased ratios of maximal intra-cavernous pressure (ICP) or total ICP to mean systolic blood pressure (MSBP) compared to those of control mice. LRG1 injection restored erection parameters in diabetic mice to almost 92% of control values (Fig. 3f). The rescue effects of LRG1 injection were significantly abrogated by shLPHN2 lentiviral infection, but not by shControl lentiviral infection (Supplementary Fig. 3a). The effects of LRG1 on erection were dose-dependent and maintained for at least 4 weeks (Supplementary Fig. 3b, c). MSBP did not differ among the experimental groups (Supplementary Tables 1–4). We also found that the LRG1-mediated restoration of erectile function was slightly attenuated in the presence of a TGF-β1-blocking antibody and was significantly decreased by further infection with shLPHN2 lentivirus (Fig. 3f). Consistently, erectile function remained intact in LRG1-Tg mice, even after multiple STZ injections, and this effect was abolished by shRNA-mediated LPHN2 knockdown (Supplementary Fig. 3d). These results indicate that intracavernous administration of recombinant LRG1 effectively ameliorates erectile dysfunction in STZ-induced diabetic mice through the LPHN2 receptor.

### The LRG1/LPHN2 Axis Exerts Dual Angiogenic and Neurotrophic Effects on the Recovery of Erectile Function in Diabetic Mice

**Fig. 4:**
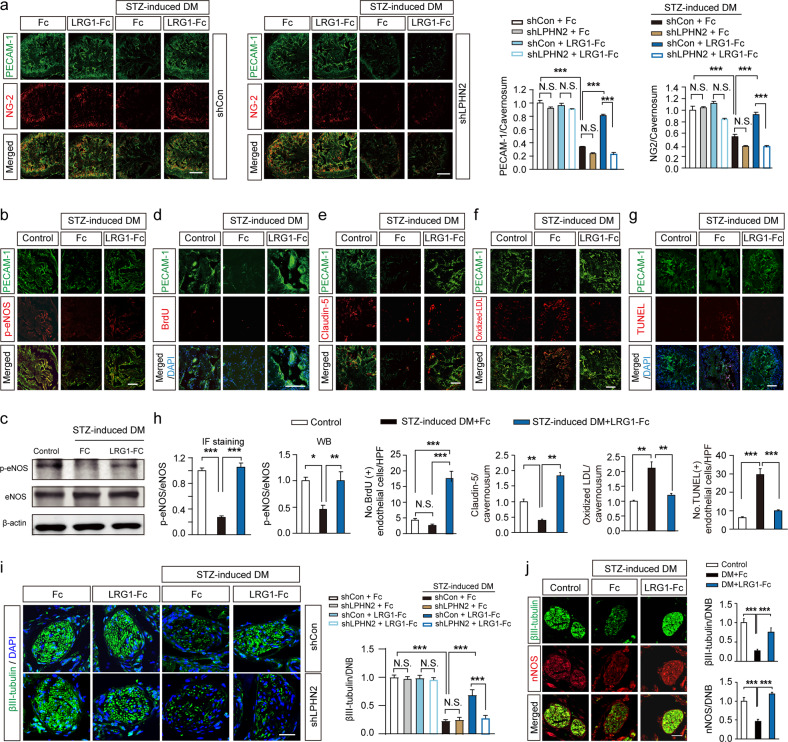
Intracavernous administration of LRG1 ameliorates vascular and neurological abnormalities via LPHN2 receptor in diabetic mouse model. **a** Immunostaining of PECAM-1 (green) and neuron-glial antigen 2 (NG2, red) in cavernous tissue from control and STZ-induced diabetic mice at 2 weeks after repeated intracavernous injections of Fc (negative control, 5 µg/20 µl) or LRG1-Fc (5 µg/20 µl) in Days 0 and 3 under shCon or shLPHN2 conditions (5 × 10^5^ TU/mouse) (left and middle). Scale bars, 100 μm. PECAM-1 and NG2 (+) areas per cavernosum were quantified using ImageJ (right, *n* = 5). **b, d, e, f** and **g** Immunostaining of p-eNOS (**b**), BrdU (**d**), Claudin-5 (**e**), oxidized-low-density lipoprotein (LDL, **f**), TUNEL (**g**) (red) and PECAM-1 (green) in cavernous tissue from control and STZ-induced diabetic mice 2 weeks after repeated intracavernous injections of Fc or LRG1-Fc (5 µg/20 µl) on Days 0 and 3. Nuclei were stained with DAPI (blue, **d** and **g**). Scale bars, 100 μm (scale bars, 50 μm for only **d**). **c** Representative Western blots showing p-eNOS and eNOS in whole penile tissue from control and STZ-induced diabetic mice 2 weeks after repeated intracavernous injections of Fc (negative control, 5 µg/20 µl) or LRG1-Fc (5 µg/20 µl) on Days 0 and 3. **h** Quantification of p-eNOS by immunostaining and Western blotting *(n* = 4), the number of BrdU (+) endothelial cells per high-power field (*n* = 4), claudin-5 and oxidized-LDL per cavernosum (*n* = 4), and the number of TUNEL (+) apoptotic endothelial cells per high-power field (*n* = 4). **i** and **j** Immunostaining of βIII-tubulin (green) and nNOS (red) in DNB tissue from control and STZ-induced diabetic mice 2 weeks after repeated intracavernous injections of Fc (negative control, 5 µg/20 µl) or LRG1-Fc (5 µg/20 µl) on Days 0 and 3 under shCon or shLPHN2 conditions (5 × 10^5^ TU/mouse, only for **i**). Nuclei were labeled with DAPI (blue). Scale bars, 25 µm for (**i**) and 100 μm for (**j**). βIII-tubulin or nNOS (+) areas in DNB tissue per high-power field were quantified using ImageJ (means ± SEM (*n* = 6), right). *P < 0.05; **P < 0.01; ***P < 0.001 (Student’s t test). N.S., not significant. The relative ratio of the shCon+Fc group or control group was defined as 1.

We performed immunofluorescence staining for platelet/endothelial cell adhesion molecule (PECAM)-1 and the pericyte marker neuron-glial antigen 2 (NG2) in cavernosum tissue. The results confirmed that LRG1 treatment rescued the decreased numbers of cavernous endothelial cells and pericytes in diabetic mice, and this effect was abolished when LRG1 was co-administered with shLPHN2 lentivirus (LRG1 + shLPHN2 group) (Fig. 4a). Expression levels of PECAM-1 and NG-2 were comparable between shControl and shLPHN2 lentivirus-infected wild-type mice, regardless of the presence of LRG1. In contrast, in diabetic mice, LRG1 treatment increased the phosphorylation of endothelial NO synthase (eNOS) (Fig. 4b, c), the endothelial cell proliferation (BrdU incorporation assay) (Fig. 4d, h), and the expression of the endothelial cell–cell junction protein claudin-5 (Fig. 4e, h), but decreased cavernous endothelial permeability (extravasation of oxidized LDL) (Fig. 4f, h) and apoptosis (TUNEL assay) (Fig. 4g, h). Similar to the findings regarding erectile function, cavernous endothelial cell levels and permeability were preserved in LRG1-Tg mice, even after multiple STZ injections, but were abrogated in the LPHN2-knockdown condition (Supplementary Fig. 4a). Interestingly, in STZ-induced diabetic mice, intracavernous injection of LRG1 significantly restored penile neuronal levels (βIII-tubulin) and neuronal NOS (nNOS)-containing nerve fibers, and these effects were abolished by LPHN2 knockdown (Fig. 4i, j). LRG1-Tg mice under STZ-induced diabetic conditions exhibited maintained nerve content in DNBs, similar to the cavernous endothelial cell content (Supplementary Fig. 4b). Taken together, our results indicate that exogenous LRG1 treatment profoundly restores the decreased numbers of endothelial cells in cavernosum tissue and neurofilaments at DNBs, thereby ameliorating erectile dysfunction in STZ-induced diabetic mice.

### The LRG1-LPHN2 Signaling Pathway is Responsible for Angiogenic and Neurotrophic Effects

**Fig. 5:**
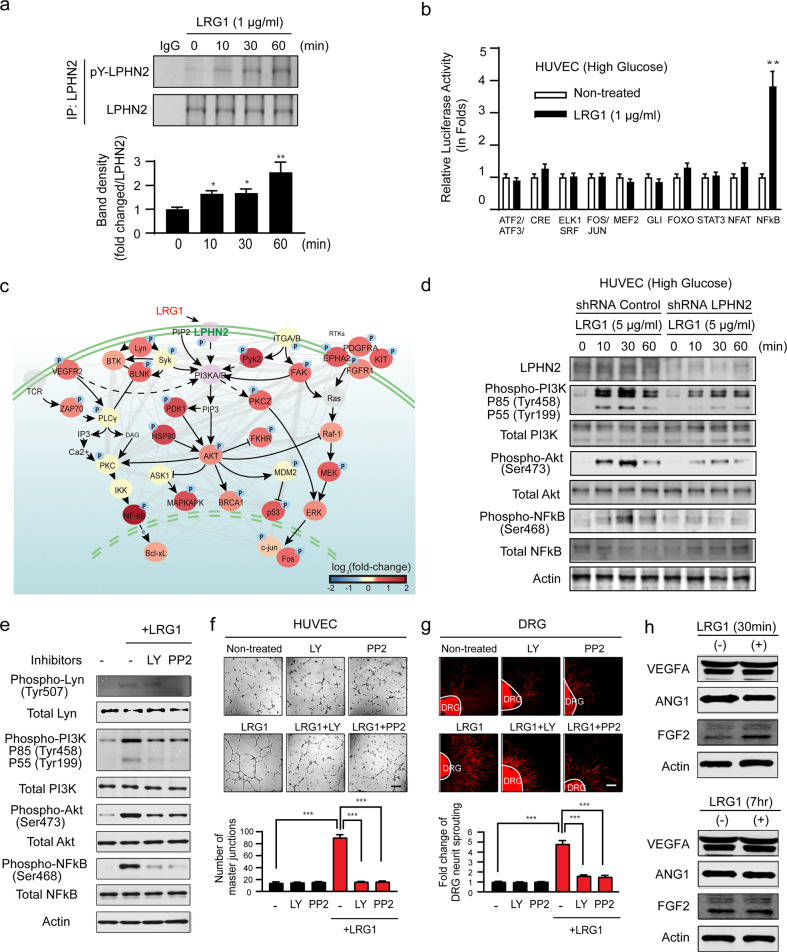
Identification of the LRG1-LPHN2 signaling pathway. **a** HUVECs were stimulated with LRG1 (1 μg/ml) for the indicated times under high-glucose conditions. Immunoprecipitants with an anti-LPHN2 antibody were analyzed by Western blotting using antibodies specific for pY-LPHN2 and LPHN2. **b** Cignal Finder GPCR signaling 10-pathway reporter array analysis of HUVECs after treatment with LRG1 (1 μg/ml). The results of dual-luciferase assays are presented as normalized relative luminescence signals (means ± SEM, *n* = 3). The relative ratio of the untreated group was defined as 1. **P < 0.01 (Student’s *t* test). **c** Network model showing interactions between the proteins with increased phosphorylation in response to LRG1. Node color indicates the increase (red) in the phosphorylation level. The color bar indicates the gradient of the log2-fold-change of phosphorylation levels by LRG1 with respect to those in untreated control conditions. Circled P on a node indicates phosphorylation of the corresponding protein. Arrows, activation; inhibition symbols, inhibition; solid arrows, direct activation; dotted arrows, indirect activation; gray lines, protein–protein interactions; and green lines, plasma membrane. **d** Western blot analysis of HUVECs stimulated with LRG1 (5 μg/ml) using the indicated antibodies. **e** Western blot analysis of HUVECs stimulated with 1 µg/ml LRG1 with or without pre-treatment with PP2 (10 μM) or LY294002 (10 μM). **f** and **g** HUVECs (f) and mouse DRG explants (g) were treated with LRG1 (1 μg/ml), LY (10 μM), PP2 (10 μM), LRG1 (1 μg/ml) + LY (10 μM), or LRG1 (1 μg/ml) + PP2 (10 μM). Top: Representative images of HUVEC tube formation (**f)** and βIII-tubulin staining in mouse DRG explants **(g**). The relative ratio of the untreated group was defined as 1. Scale bars, 100 µm. Bottom: Master junctions (*n* = 4) (**f**) and βIII-tubulin–immunopositive axon lengths in DRGs (**g**) were quantified using ImageJ, and the results are presented as the means ± SEM (*n* = 4). **h** Western blot analysis of HUVECs stimulated with 1 µg/ml LRG1 for 30 min or 7 hr using antibodies specific for angiogenic factors (VEGFA, angiopoietin-1, FGF2).

Previous proteomic screens have identified two tyrosine residues in LPHN2 (Tyr1406 and Tyr1421) as candidate phosphorylation sites, although the significance of LPHN2 phosphorylation is unknown^41^. Therefore, we next examined whether LPHN2 is phosphorylated upon LRG1 binding under high-glucose conditions. Immunoprecipitation of LPHN2 and subsequent immunoblotting with anti-phospho-tyrosine antibody demonstrated that LRG1 treatment stimulated LPHN2 phosphorylation within 10 min. These results suggest that LRG1 binding can induce LPHN2-mediated intracellular signaling (Fig. 5a).

To examine the LRG1/LPHN2-mediated intracellular signaling pathway responsible for promoting angiogenic and neurotrophic effects, we first used a Cignal Finder GPCR Signaling 10-Pathway Reporter Array in HUVECs upon LRG1 treatment. Of the 10 pathways tested, only nuclear factor kappa-light-chain-enhancer of activated B cells (NF-κB) was activated by LRG1 treatment under hyperglycemic culture conditions (Fig. 5b). To systematically investigate the signaling components affected by LRG1, we performed phosphorylation profiling of 363 key signaling proteins in HUVECs after LRG1 treatment. Among the 688 phosphorylation sites on the array, 102 exhibited phosphorylation increases (51 sites) or decreases (51 sites) of >25% upon LRG1 treatment (Supplementary Fig. 5). We next built a signal network model describing the interactions between these proteins (Fig. 5c). This network model predicted that LRG1 can induce phosphorylation of NF-κB p65 and its upstream kinase AKT, and this prediction was confirmed by Western blot analysis of HUVECs (Fig. 5d). Western blot analysis also revealed phosphorylation of the upstream AKT kinase PI3K, which was not detected by the phosphor-array. Phosphorylation levels of PI3K, AKT, and NF-κB p65 increased 10–30 min after LRG1 treatment under high-glucose conditions and declined after 60 min (Fig. 5d). HUVECs with shRNA-mediated LPHN2 knockdown exhibited reduced phosphorylation of PI3K, AKT, and NF-κB p65, even upon LRG1 treatment (Fig. 5d). Moreover, LRG1-induced phosphorylation of Lyn was markedly decreased by PP2, a Lyn kinase (Src kinase family) inhibitor, and both PP2 and LY294002 (PI3K inhibitor) substantially suppressed PI3K, AKT and NF-κB p65 phosphorylation even in the presence of LRG1 (Fig. 5e). Consequently, both PP2 and LY294002 inhibited tube formation in HUVECs and axonal sprouting from DRG explants, even in the presence of LRG1 (Fig. 5f, g). Finally, we investigated whether LRG1 exerts its angiogenic effects directly or indirectly. The protein levels of typical angiogenic factors were analyzed, and there were no differences in the levels of VEGFA, angiopoietin-1 (ANG1) or fibroblast growth factor 2 (FGF2) between untreated and LRG1–treated HUVECs (Fig. 5h), indicating that endothelial cell proliferation and migration mainly result from the direct effects of LRG1/LPHN2-mediated signaling through Lyn/PI3K/AKT/NF-κB p65. Collectively, these results imply that PI3K, AKT, and NF-κB p65 signaling constitutes the key signaling pathway for LRG1/LPHN2-mediated angiogenesis and neurite outgrowth under hyperglycemic conditions.

## Discussion

In this study, we found that even in the absence of TGF-β, LRG1 can induce endothelial tube formation and migration. Therefore, we postulated that LRG1 may also mediate angiogenesis through an alternative receptor that is independent of TGF-β signaling. Using a ligand–receptor capture (LRC)-based proteomics approach and comprehensive biochemical and cellular analysis, we found that LPHN2 is a novel TGF-β-independent receptor of LRG1. Among LPHN family members (LPHN1–3), LPHN1 and LPHN3 are predominantly expressed in the brain and play critical roles in growth cone migration and synapse formation by binding to adhesion ligands, including teneurin, neurexins, and fibronectin leucine-rich repeat transmembrane proteins (FLRTs)^36^. On the other hand, LPHN2 is widely expressed in various tissues and reportedly participates in synapse formation, epithelial–mesenchymal transition (EMT), cardiac development, and vascular morphogenesis^35,42–45^. However, little is known about specific ligands of LPHN2 or the downstream signaling pathways and molecular mechanisms underlying the cellular functions of LPHN2. Our results showed that LRG1 specifically and directly binds to the LPHN2 Olf domain but not to LPHN1 or LPHN3.

Blood vessels and nerve fibers exhibit comparable patterning and, in some locations, track alongside each other^46^, suggesting potential similarities between the mechanisms involved in wiring neural and vascular networks. Accordingly, various factors known as angioneurins (e.g., VEGF, angiopoietin, NGF, BDNF, FGF2, and HGF) have dual roles in vascular and neuronal development, and their malfunction can contribute to both neurological and vascular disorders^47^. Our results demonstrated that LRG1 can function as an angioneurin by directly binding and activating LPHN2. The binding of LRG1 to LPHN2 promoted endothelial cell proliferation and tube formation, microvessel sprouting from aortic rings, and neurite outgrowth from the DRG and MPG through PI3K-AKT-NF-κB p65 phosphorylation. NF­κB is a key regulator of innate and adaptive immune responses, which can be activated by the cytokines TNFα and IL-1 and by bacterial components, such as lipopolysaccharide^48^. Other angioneurins, such as VEGF, NT3, and BDNF, can also activate NF-κB to induce vascular endothelial responses and axonal and dendritic growth^49,50^. Although the NF-κB target gene associated with LRG1/LPHN2-mediated angiogenesis and neurite outgrowth remains to be identified, we determined that the PI3K-AKT-NF-κB p65 signaling pathway is entirely independent of TGF-β. In future studies, it will be interesting to determine whether LRG1 can simultaneously or exclusively activate LPHN2-mediated and TGF-β receptor-mediated signaling in endothelial and neuronal cells under pathophysiological conditions.

Erectile dysfunction is common in patients with DM. Despite its substantial negative impacts on quality of life, ED is often undiagnosed in the clinic and remains undertreated. Among diabetic men, the main etiologies of ED are endothelial dysfunction, autonomic neuropathy, and smooth muscle dysfunction^1^. There is an unmet need for therapies that can cure both diabetic vascular and neuronal complications, highlighting the urgency of developing novel treatment options. Several angioneurins, including VEGF, dickkopf2, COMP-Ang1, neurotrophin-3 (NT3), and brain-derived neurotrophic factor (BDNF), have been investigated as potential treatment options for diabetic ED^4–8^. Although the detailed mechanisms are not yet clear, we detected increased LPHN2 expression in the penile tissues of STZ-induced diabetic mice and human diabetic patients. Importantly, intracavernous administration of LRG1 in diabetic mice elicited a wide range of beneficial therapeutic effects through its interaction with LPHN2, including increases in eNOS phosphorylation, endothelial proliferation, endothelial cell–cell junctions, and the proportion of nNOS-containing nerve fibers, as well as decreases in cavernous endothelial permeability and apoptosis (Figs. 3 and 4). These benefits of exogeneous LRG1 ameliorated vascular and neurological abnormalities in the penile tissue of diabetic mice, ultimately leading to the restoration of erectile function. Notably, LRG1 treatment in normal mice did not cause any vascular or neuronal alterations, regardless of the presence of LPHN2, suggesting that intracavernous injection of LRG1 will have limited potential side effects. This preclinical evidence indicates that local administration of LRG1 could be a promising therapeutic strategy for the treatment of diabetic vascular and neuronal complications, such as diabetic erectile dysfunction and foot ulcers. The STZ-induced type 1 diabetic mouse model has been widely used and has contributed to substantial advancements in the study of ED^51^. However, in the clinical setting, only 10% of DM cases are type 1 DM, while 90% of cases are type 2 DM^52^. Thus, there remains a need for further investigation of the therapeutic effects of LRG1 on diabetic ED using animal models of ED associated with type 1 and/or 2 diabetes.

LRG1 is known to play important roles in diabetic diseases. Patients with type 2 diabetes exhibit elevated plasma LRG1 levels, which seems to be related to the occurrence and progression of diabetic nephropathy^53,54^ and proliferative diabetic retinopathy^20,55^. However, several recent studies have shown that LRG1 may be a potential therapeutic option for chronic diabetic wounds, diabetic keratopathy, and obesity^22–24^. Overall, these findings suggest that LRG1 is a multifunctional protein with context-dependent effects. Indeed, LRG1 is reportedly involved in neovascularization, epithelial–mesenchymal transition (EMT), neutrophil activation, re-epithelialization, and nerve regeneration^22,23,25,56,57^. Additionally, LPHN2 expression is highly increased during the differentiation of pluripotent stem cells into cardiac progenitor cells and cardiomyocytes, and *Lphn2*^−/−^ embryos exhibit serious defects in right ventricle, right atrium, and outflow tract development^43^, suggesting that the LRG1/LPHN2 signaling axis may also be involved in vascular and neuronal development.

Based on our current and previous findings, we propose a model suggesting that high circulating LRG1 in serum can only exert its cellular functions at specific sites with increased expression of LPHN2 and/or TGF-β, although the role of highly abundant serum LRG1 is not yet clear. Conversely, this model predicts that under pathological conditions, excess LRG1 expression or overactivation of the LRG1/LPHN2 axis may cause chronic inflammation via NF-κB activation^21^. Thus, LRG1 can be viewed as a double-edged sword, similar to growth factors in cancers and cytokines in sepsis. It remains to be clarified whether increased LRG1 under pathological conditions causes pathological abnormalities or is a consequence of compensatory effects during recovery in pathologically damaged tissues. Since LRG1 has long been considered a promising biomarker for various pathologies, including inflammation, neurodegenerative disease, heart failure, and several types of cancer^10–18^, further studies should investigate other roles of LRG1 in these diseases.

## Supplementary information

Supplemental Information

## Data Availability

All study data are included in the article and/or Supplemental Information. The phospho array data have been deposited in Gene Expression Omnibus (GSE160005).
