# Supplementary material for: Latrophilin-2 is a novel receptor of LRG1 that rescues vascular and neurological abnormalities and restores diabetic erectile function
Source: Exp Mol Med. 2022 May 13;54(5):626–38. doi: 10.1038/s12276-022-00773-5 (PMC9166773; doi:10.1038/s12276-022-00773-5)
Supplement: Supplementary file 1 — Supplemental Information [file 12276_2022_773_MOESM1_ESM.pdf]

Supplementary Information

Latrophilin-2 is a novel receptor of LRG1 that rescues vascular and neurological abnormalities and restores diabetic erectile function

*Corresponding Authors

Ho Min Kim, Email: hm_kim@kaist.ac.kr; Ji-Kan Ryu, Email: rjk0929@inha.ac.kr; Jun-Kyu Suh, Email: jksuh@inha.ac.kr

This PDF file includes:

Supplementary Figure
Supplementary Table
Supplementary References

Supplementary Figures

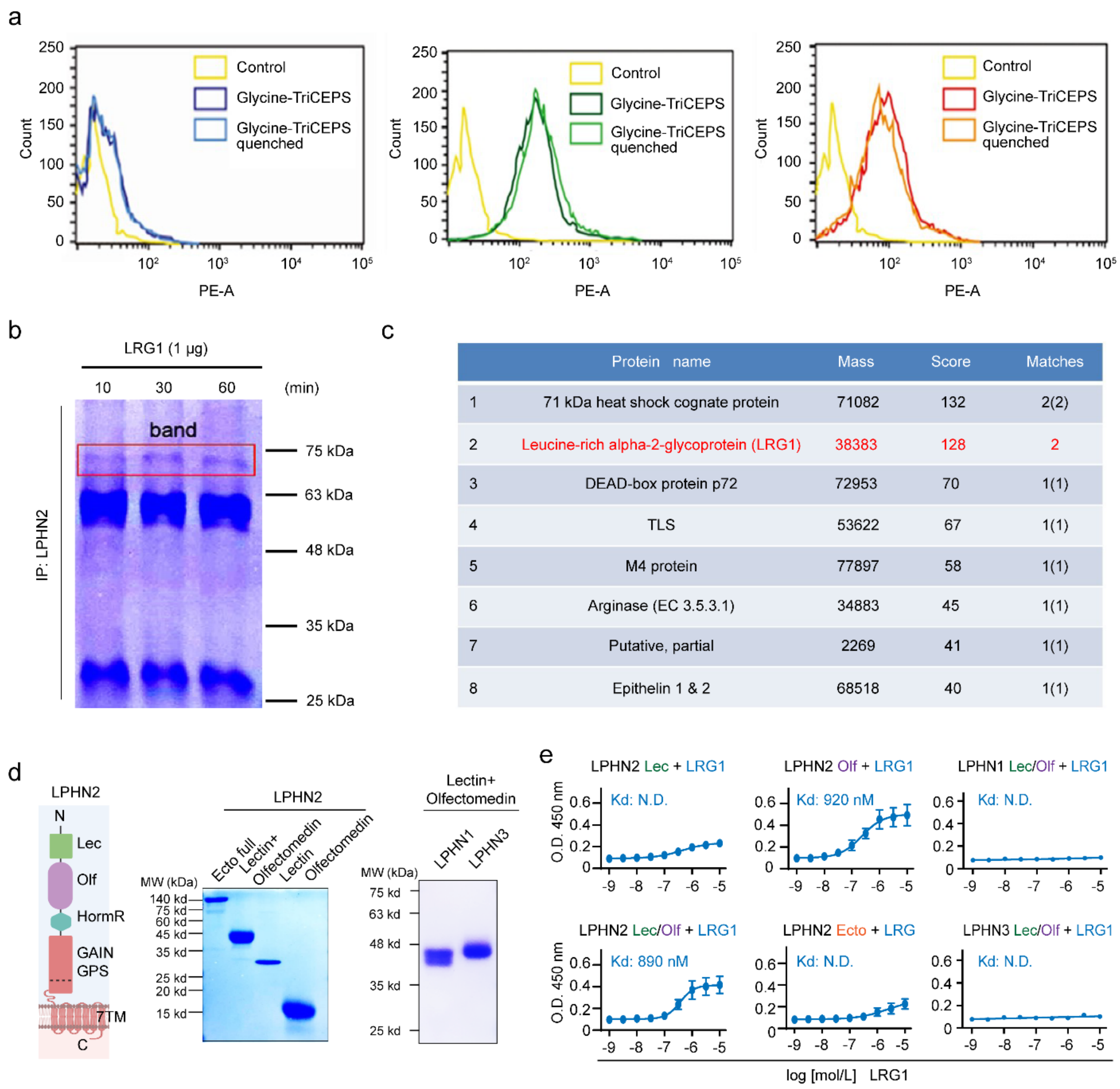

	Protein name	Mass	Score	Matches
1	71 kDa heat shock cognate protein	71082	132	2(2)
2	Leucine-rich alpha-2-glycoprotein (LRG1)	38383	128	2
3	DEAD-box protein p72	72953	70	1(1)
4	TLS	53622	67	1(1)
5	M4 protein	77897	58	1(1)
6	Arginase (EC 3.5.3.1)	34883	45	1(1)
7	Putative, partial	2269	41	1(1)
8	Epithelin 1 & 2	68518	40	1(1)

Supplementary Fig. 1 Identification of a TGF-β-independent receptor for LRG1.

a Ligands, including the negative control glycine, positive control transferrin and LRG1, were conjugated with the biotin-containing TriCEPS reagent using its NHS ester functionality. Binding of TriCEPS-coupled ligands to cell surface receptors was detected by FITC-streptavidin and analyzed by FACS. **b** HUVECs were treated with LRG1 (1 μg/ml) at the time intervals indicated, then LPHN2 was immunoprecipitated (IP) from whole-cell lysates, resolved on SDS-PAGE gels, and stained with Coomassie Blue solution. **c** Identification of LPHN2-interacting proteins by liquid

chromatography tandem mass spectrometry (LC-MS/MS) analysis. Excised gel bands for LC-MS/MS analysis (~70 kDa) are indicated in Figure S1B. Proteins identified by LC-MS/MS analysis are listed according to the top matching peptides. **d** Schematic domain architecture of LPHN2 (left, Abbreviations: Lec, lectin; Olf, olfactomedin-like; HomoR, hormone receptor motif; GAIN/GPS, GPCR autoproteolysis-inducing/GPCR proteolysis site). Purified recombinant human LPHN2 ectodomain variants (Lectin domain, residues F26-Q95 (Lec); Olfactomedin-like domain, residues V135-P394 (Olf); Lectin-Olfactomedin-like domain, residues F26-P394 (Lec/Olf); and Lectin-Olfactomedin-like-GAIN/GPS domain, residues F26-R796 (Ecto)) and Lectin-Olfactomedin-like domain (Lec/Olf) of human LPHN1 and LPHN3 were analyzed by SDS-PAGE and Coomassie Blue staining (middle and right). **e** Binding affinity of LRG1 to the LPHN2 ecto-domains (Lec, Olf, Lec/Olf or Ecto-full domain) or Lec/Olf of LPHN1 and LPHN3 were determined by solid-phase binding assay. LPHN2 extracellular domain variants (Lec, Olf, GAIN/GPS domain; 100 nM) were added to MaxiSorp 96-well plates (Nunc) and incubated for 1 hour at room temperature. Wells were washed twice with PBS and then incubated with 1% bovine serum albumen (BSA) for 2 hours. After blocking, varying amounts (1, 10, 100 nM and 1, 10, 100 □ M) of native were added to 96-well plates coated with the indicated LPHN2 extracellular domain variants. LRG1 bound to coated proteins was detected by ELISA using an anti-LRG1 antibody (Cat# sc517443, Santa Cruz) and peroxidase-conjugated anti-mouse secondary antibody (Cat# 62-6520, Thermo Fisher Scientific).

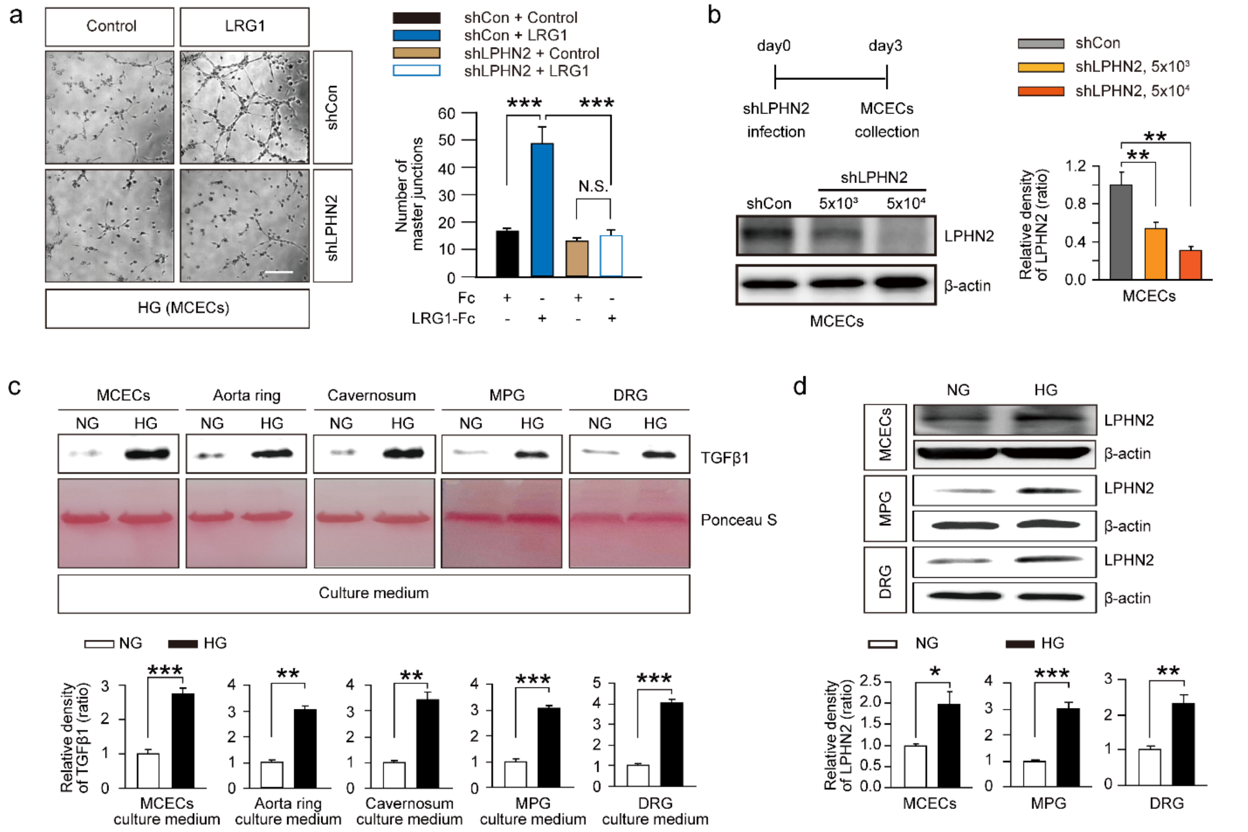

Supplementary Fig. 2 LRG1/LPHN2-mediated endothelial cell tube formation and expression of TGFβ and LPHN2 under hyperglycemia.

a Tube-formation assay. MCECs under high-glucose (HG) conditions were incubated with PBS (negative control) or LRG1 (1 μg/ml) for 72 hours, and then assayed for tube formation. Control (shCon) and LPHN2-knockdown (shLPHN2) lentiviruses were added to culture medium at 5 x10^4 transduction units (TU)/ml. Representative images of tube formation (left). Scale bars, 100 μm. Master junctions were quantified using Image J and the results are presented as means ± SEM (n = 4, right). **b** MCECs were infected with lentivirus containing control shRNA (shCon) or shRNA targeting LPHN2 (shLPHN2) at two different doses (5 x10^3 TU and 5 x10^4 TU/ml culture medium) for at least 72 hours. Cells was harvested at 3 days after infection. Left: Representative Western blots for LPHN2 from MCECs (top) infected with shCon or shLPHN2 lentivirus. Right: Normalized band intensity values were quantified using Image J and the results are presented as means ± SEM (n = 4). The relative ratio of the shCon group was defined as 1. **c** Increased TGFβ1 levels in culture medium under HG conditions. Representative Western blots for TGFβ1 in conditioned medium from MCECs, aorta ring tissue, cavernosum tissue, MPG and DRG tissue under NG and HG conditions (top). Normalized band intensity values were quantified using Image J and the results are presented as means ± SEM (n = 4, bottom). Ponceau S used as an internal loading

control. **d** Increased LPHN2 expression in MCECs, MPG, and DRG under NG or HG conditions. Representative Western blots for LPHN2 (top). Normalized band intensity values quantified by Image J and presented as means ± SEM (*n* = 4, bottom). (**c and d**) The relative ratio of the NG control group was defined as 1. $^{**}P < 0.01$; $^{***}P < 0.001$ (Student's t test).

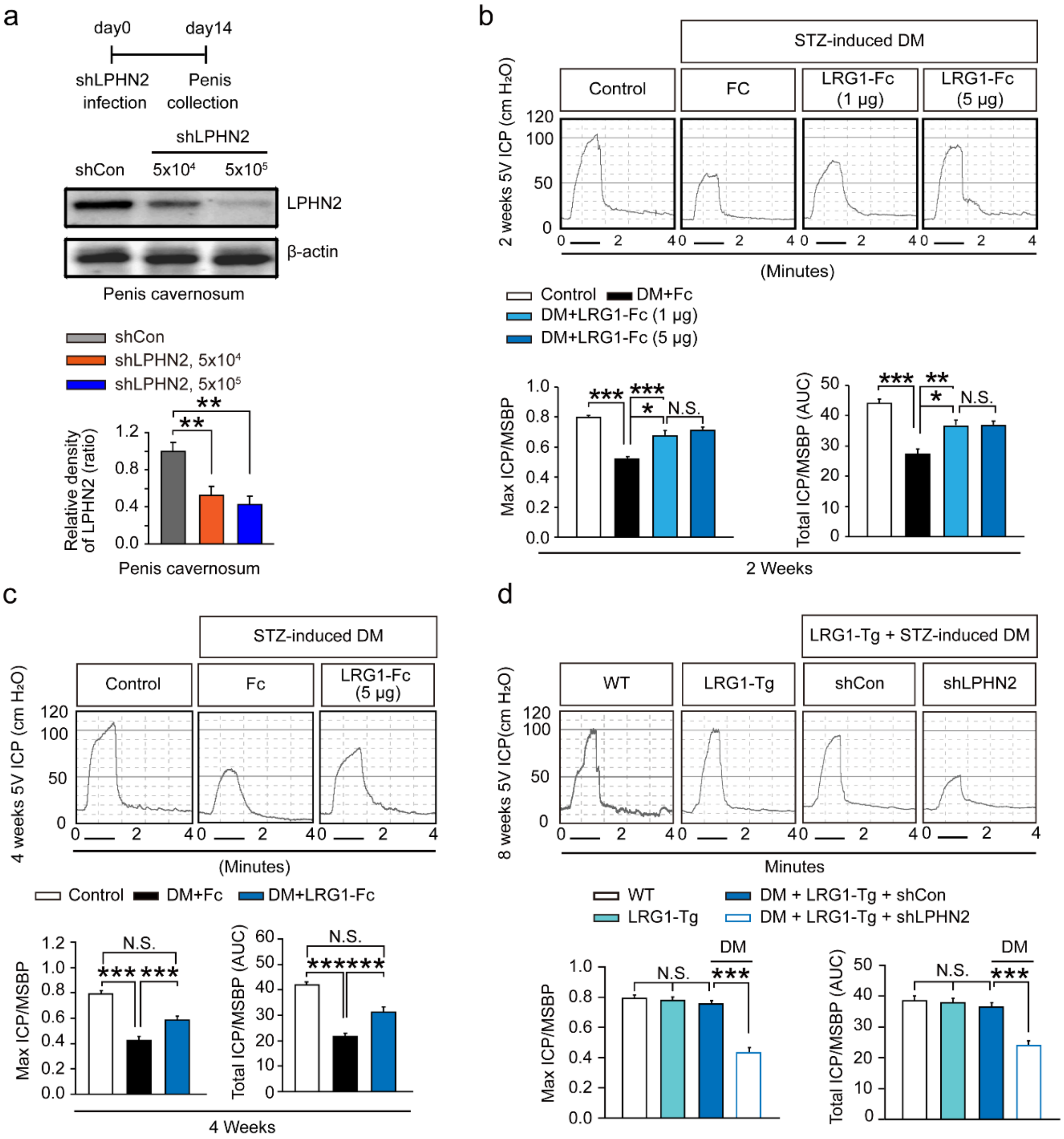

Supplementary Fig. 3 Exogenous recombinant LRG1 or endogenously overexpressed LRG1 ameliorates diabetic erectile dysfunction.

a LPHN2 knockdown with lentivirus. Mouse penis was infected with scramble shRNA control (shCon) and LPHN2 knockdown shRNA (shLPHN2) lentivirus at two different doses (5×10^4 TU and 5×10^5 TU/ml culture medium) for at least 72 hours. Tissue was harvested at 14 days after infection. Representative Western blots for LPHN2 from mouse penis cavernosum tissues infected with shCon or shLPHN2 lentivirus (top). Normalized band intensity values were quantified using Image J and the results are presented as means ± SEM (bottom, n = 4). The relative ratio of the

shCon group was defined as 1. **b and c** Representative intracavernous pressure (ICP) responses in the control and STZ-induced diabetic mice at 2 weeks **(b, top)** and 4 weeks **(c, top)** after repeated intracavernous injections of Fc (negative control, 10 µg/20µl), and LRG1-Fc (1 µg/20µl or 5 µg/20µl, respectively) at day 0 and 3. **d** Analysis of erectile function in STZ-induced diabetic LRG1-Tg mouse. Representative intracavernous pressure (ICP) responses at 8 weeks in the LRG1-Tg mice receiving STZ injection under scramble shRNA control (shCon) or LPHN2 knockdown shRNA (shLPHN2) condition (1×10^5 TU/mouse) (top). *$P < 0.05$; **$P < 0.01$; ***$P < 0.001$ (Student's t test). N.S., not significant. The cavernous nerve was stimulated at 5 V. The stimulus interval is indicated by a solid bar. Ratios of mean maximal ICP and total ICP (area under the curve) to mean systolic blood pressure (MSBP) were calculated for each group (**b, c and d**, bottom, $n = 5$)

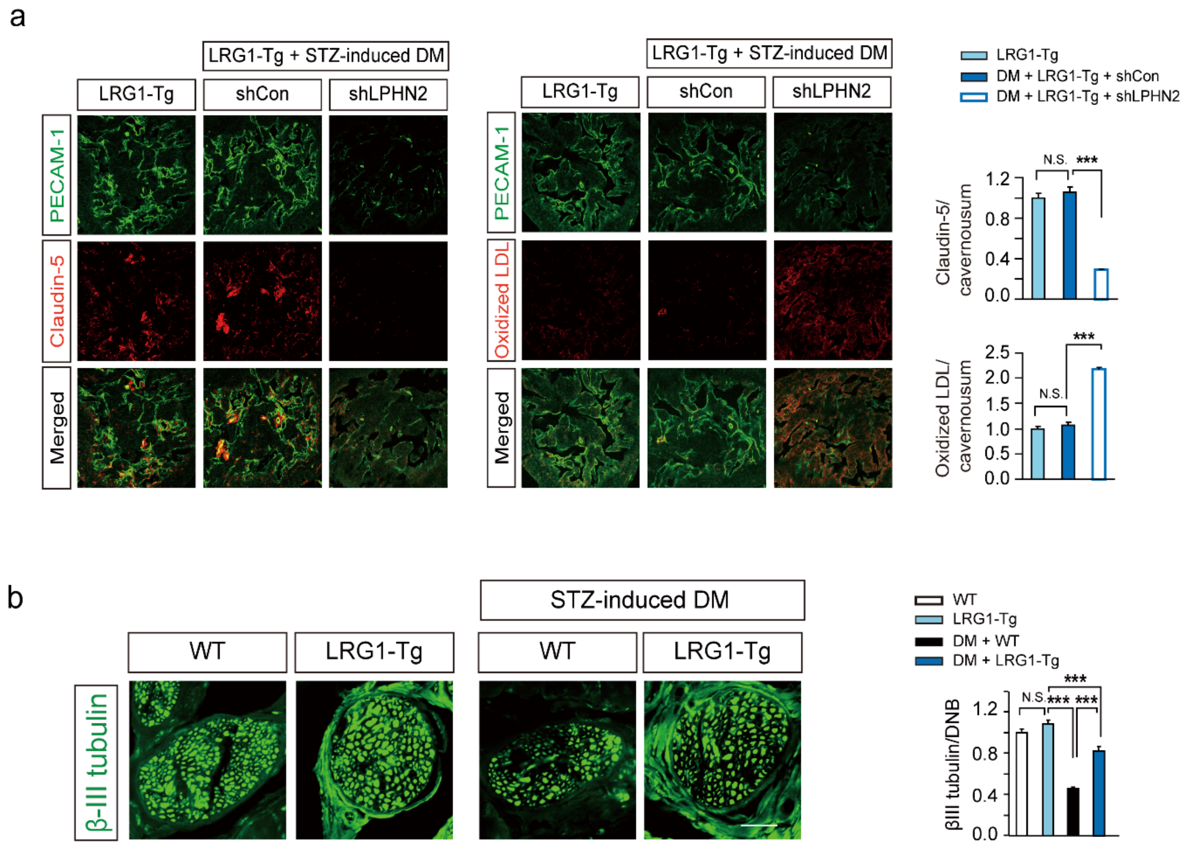

Supplementary Fig. 4 Angiogenic and neurotrophic effect of LRG1 in STZ-induced diabetic LRG1-Tg mouse.

a Immunostaining of Claudin-5 (left, red), oxidized-LDL (middle, red) and PECAM-1 (green) in cavernous tissue from LRG1-Tg mice and STZ-induced LRG1-Tg mice with shCon or shLPHN2 lentivirus (5 × 10^5 TU/mouse). Quantification of Claudin-5 (right, top) and Oxidized LDL (right, bottom) expression using Image J (n = 4). The relative ratio of the LRG1-Tg group was defined as 1. Scale bars, 100 µm. **b** βIII-Tubulin (green) immunostaining in DNB from wild-type (WT) control and LRG1-Tg mice with or without STZ injections for 8 weeks (left). The βIII-tubulin (+) area in the dorsal nerve bundle was quantified using Image J and the results are presented as means ± SEM (right, n = 4). Scale bars, 25 µm. The relative ratio of the WT group was defined as 1. ***$P < 0.001$ (Student's t test). N.S., not significant.

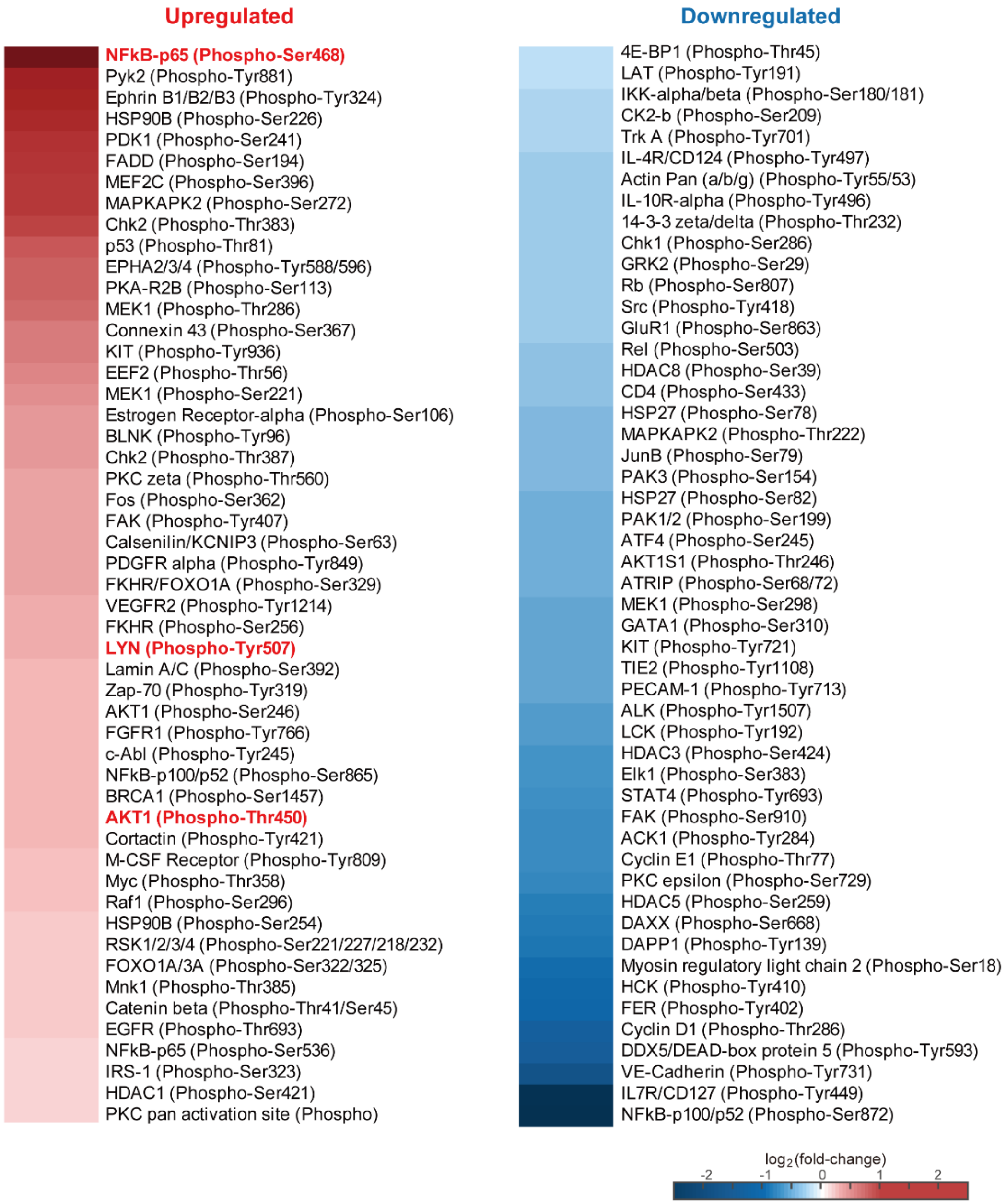

Supplementary Fig. 5 Phosphorylation profiling of HUVEC upon treatment of LRG1.

Altered protein and phosphorylation levels of the proteins after LRG1 treatment. Colors indicate the increase (red) and decrease (blue) in the levels by LRG1. Color bar, the gradient of the log2-fold-change of protein or phosphorylation levels by LRG1 with respect to those in non-treated control conditions. Whether protein and phosphorylation levels are displayed is indicated by “Ab-” and “Phospho-” in the labels within parenthesis, respectively. NF-κB and its upstream phosphorylated proteins (LYN and AKT1) are denoted in red. For network analysis, we first

identified the proteins showing the more than 25% increases in their phosphorylation levels by LRG1 treatment. We then obtained protein-protein interactions (PPIs) for these phosphorylated proteins from the ten interactome databases including BioGRID[1], HuRI[2], IntAct[3], HitPredict[4], IID[5], MINT[6], DIP[7], HPRD[8], HTRIdb[9], and STRING[10]. Using these PPIs, we constructed a network model describing the interactions between the identified phosphorylated proteins. A number of proteins that can help understand activation of LRG1-associated signaling pathways were added to the network model. In the network model, we arranged the phosphorylated proteins based on their localizations and activation/repression information obtained from KEGG pathway database[11]

Supplementary Tables

Supplementary Table 1. Physiologic and metabolic parameters. 2 weeks after treatment of LRG1-Fc.

	Control	STZ-induced diabetic mice			
		Fc	LRG1-Fc (1 µg/20 µl)	LRG1-Fc (5 µg/20 µl)	LRG1-Fc (10 µg/20 µl)
**Body weight (g)**	33.1±1.3	23.4±0.6*	22.4±0.3*	23.1±0.7*	23.4±0.5*
**Fasting glucose (mg/dl)**	105.0±3.7	389.7±41.5*	245.9±8.9*	327.1±18.7*	291±19.8*
**Postprandial glucose (mg/dl)**	145.6±7.4	532.7±22.2*	487.1±14.6*	534.1±19.8*	498.4±31.9*
**MSBP (mm Hg)**	100.2±1.9	102.8±1.7	100±3.2	102.8±1.5	101.6±1.2

Values are the means ± SEM for n = 10 animals per group. **P* < 0.05 vs. Control group. STZ, streptozotocin; MSBP, mean systolic blood pressure.

Supplementary Table 2. Physiologic and metabolic parameters. 4 weeks after treatment of LRG1-Fc.

	Control	STZ-induced diabetic mice	
		Fc	LRG1-Fc (5 µg/20 µl)
**Body weight (g)**	36.3±1.5	21.5±0.9*	24±2.0*
**Fasting glucose (mg/dl)**	110.0±11.5	462.4±83.8*	495.9±57.3*
**Postprandial glucose (mg/dl)**	177.7±11.1	594.1±10.7*	557.0±41.2*
**MSBP (mm Hg)**	127.2±7.5	126.4±9.1	132.7±8.7

Values are the means ± SEM for n = 7 animals per group. **P* < 0.05 vs. Control group. STZ, streptozotocin; MSBP, mean systolic blood pressure.

Supplementary Table 3. Physiologic and metabolic parameters.

	2 weeks after treatment of indicated protein together with shCon lentivirus				
	Fc	LRG1-Fc	STZ-induced diabetic mice		
			Fc	LRG1-Fc	LRG1-Fc+Anti-TGFβ1
**Body weight (g)**	35.7±0.9	36.5±0.6	25.4±0.5*	24.9±0.8*	24.2±0.8*
**Fasting glucose (mg/dl)**	108.0±2.3	103.4±1.7	392.8±25.9*	399.4±29.5*	412.8±21.9*
**Postprandial glucose (mg/dl)**	172.8±4.9	164.6±4.9	519.0±29.8*	543.2±20.3*	559.2±19.7*
**MSBP (mm Hg)**	102.4±1.4	103.8±2.2	102.6±1.1	104.8±1.9	107.4±3.3
	**2 weeks after treatment of indicated protein together with shLPHN2 lentivirus**				
	Fc	LRG1-Fc	STZ-induced diabetic mice		
			Fc	LRG1-Fc	LRG1-Fc+Anti-TGFβ1
**Body weight (g)**	34.7±0.5	36.7±0.5	22.7±0.4*	24.9±0.7*	22.2±0.9*
**Fasting glucose (mg/dl)**	103.8±1.9	103.4±2.2	347.4±18.3*	353.2±34.4*	400.2±28.7*
**Postprandial glucose (mg/dl)**	165.8±4.1	162.0±14.3	567.6±21.6*	587.4±8*	580±14.4*
**MSBP (mm Hg)**	98.8±3	105.0±2.5	102.8±1.9	104.2±2.4	106.8±3.0
Values are the means ± SEM for n = 6 animals per group. **P* < 0.05 vs. Fc or LRG1-Fc group. STZ, streptozotocin; MSBP, mean systolic blood pressure.					

Supplementary Table 4. Physiologic and metabolic parameters.

	8 weeks after induction of diabetes with STZ in LRG1-Tg mice			
	WT	LRG1-Tg	STZ-induced diabetic mice	
			LRG1-Tg+shCON	LRG1-Tg+shLPHN2
**Body weight (g)**	36.4±0.8	33.1±0.3	23.9±0.7*	22.7±0.7*
**Fasting glucose (mg/dl)**	103.2±3.1	114.0±2.8	436.0±15.0*	402±33.1*
**Postprandial glucose (mg/dl)**	153.4±5.2	199.6±11.9	575.8±11.6*	566.6±18.5*
**MSBP (mm Hg)**	105.0±1.6	115.0±1.6	111.0±3.3	116.0±2.4
Values are the means ± SEM for n = 5 animals per group. **P* < 0.05 vs. Lrg1-Tg or WT group. STZ, streptozotocin; MSBP, mean systolic blood pressure.				

Supplementary References

1 Stark, C. *et al.* BioGRID: a general repository for interaction datasets. *Nucleic Acids Res.* **34**, D535-539 (2006).

2 Luck, K. *et al.* A reference map of the human binary protein interactome. *Nature* **580**, 402-408 (2020).

3 Hermjakob, H. *et al.* IntAct: an open source molecular interaction database. *Nucleic Acids Res.* **32**, D452-455 (2004).

4 Patil, A., Nakai, K. & Nakamura, H. HitPredict: a database of quality assessed protein-protein interactions in nine species. *Nucleic Acids Res.* **39**, D744-749 (2011).

5 Kotlyar, M., Pastrello, C., Malik, Z. & Jurisica, I. IID 2018 update: context-specific physical protein-protein interactions in human, model organisms and domesticated species. *Nucleic Acids Res.* **47**, D581-D589 (2019).

6 Chatr-aryamontri, A. *et al.* MINT: the Molecular INTeraction database. *Nucleic Acids Res.* **35**, D572-574 (2007).

7 Xenarios, I. *et al.* DIP, the Database of Interacting Proteins: a research tool for studying cellular networks of protein interactions. *Nucleic Acids Res.* **30**, 303-305 (2002).

8 Keshava Prasad, T. S. *et al.* Human Protein Reference Database--2009 update. *Nucleic Acids Res.* **37**, D767-772 (2009).

9 Bovolenta, L. A., Acencio, M. L. & Lemke, N. HTRIdb: an open-access database for experimentally verified human transcriptional regulation interactions. *BMC Genomics* **13**, 405 (2012).

10 Szklarczyk, D. *et al.* STRING v10: protein-protein interaction networks, integrated over the tree of life. *Nucleic Acids Res.* **43**, D447-452 (2015).

11 Kanehisa, M. & Goto, S. KEGG: kyoto encyclopedia of genes and genomes. *Nucleic Acids Res.* **28**, 27-30 (2000).
